# Expanding the scope of shared decision-making in vascular access planning for hemodialysis: a case for interprofessional collaboration

**DOI:** 10.1186/s12882-025-04261-6

**Published:** 2025-07-01

**Authors:** Andrea Yu-Ling Liu, Mary Hammes, Peter Angelos, Neil Kondamuri, Lauren Harriett, Ashley Suah, Ted Albert Skolarus

**Affiliations:** 1 Department of Surgery, University of Chicago, 5841 S Maryland Ave, Chicago, IL 60637 USA; 2 MacLean Center for Clinical Medical Ethics, University of Chicago, 5841 S Maryland Ave, Chicago, IL 60637 USA; 3 Department of Medicine, University of Chicago, 5841 S Maryland Ave, Chicago, IL 60637 USA; 4 Department of Family Medicine, University of Chicago, 5841 S Maryland Ave, Chicago, IL 60637 USA

**Keywords:** Kidney failure, Vascular access planning, Hemodialysis, Shared decision-making, Implementation, Interprofessional shared decision-making, Interprofessional collaboration, Multidisciplinary team

## Abstract

Vascular access planning guidelines have shifted from a fistula-centered to a patient-centered approach. We advocate for expansion of the shared decision-making dyad between the nephrology team and patient to include other vascular access planning stakeholders. We propose earlier and consistent interprofessional collaboration facilitates more constructive discussions that could improve outcomes.

## Main text

Vascular access planning is critical to the care of patients with kidney failure requiring hemodialysis. Under the 2003 *Fistula First Initiative*, guidelines recommended arteriovenous (AV) fistula formation as optimal access. This one-size-fits-all approach was based on a large body of observational data associating AV fistulas with improved outcomes compared to indwelling vascular catheters (e.g., lower rates of infection and hospitalization, longer rates of durability, and improved survival) [1]. The belief that AV fistulas are associated with better outcomes continues to be reinforced by quality measures and dialysis center reimbursement models grounded in the prevalence of AV fistula use in patients on hemodialysis, at least in the United States (US). The legacy effects of this initiative continue to impact planning, practice, and payment for dialysis access.

The Kidney Disease Outcomes Quality Initiative (KDOQI) updated their Clinical Practice Guideline for Vascular Access in 2019, resulting in a fundamental shift from the “fistula first, catheter last” approach to one that aims to identify the “right access, in the right patient, at the right time, for the right reasons.” [1] The revised guidelines recommend an individualized approach to vascular access planning guided by shared decision-making. Through collaborative deliberation, shared decision-making facilitates a discussion of the risks and benefits of treatment options (i.e., all vascular access possibilities) and helps patients and providers determine a treatment plan that incorporates the patient’s preferences and values. This shift was based, in part, on limitations of the observational data describing vascular access outcomes and concerns that superiority of fistulas may be overestimated when compared to central venous catheters. For example, studies comparing fistula and graft patency tended to exclude abandoned fistulas or those with primary failure [1, 2]. Furthermore, the fistula-centered approach fails to consider that surgical creation of a fistula might not align with the patient’s goals of care.

**Fig. 1:**
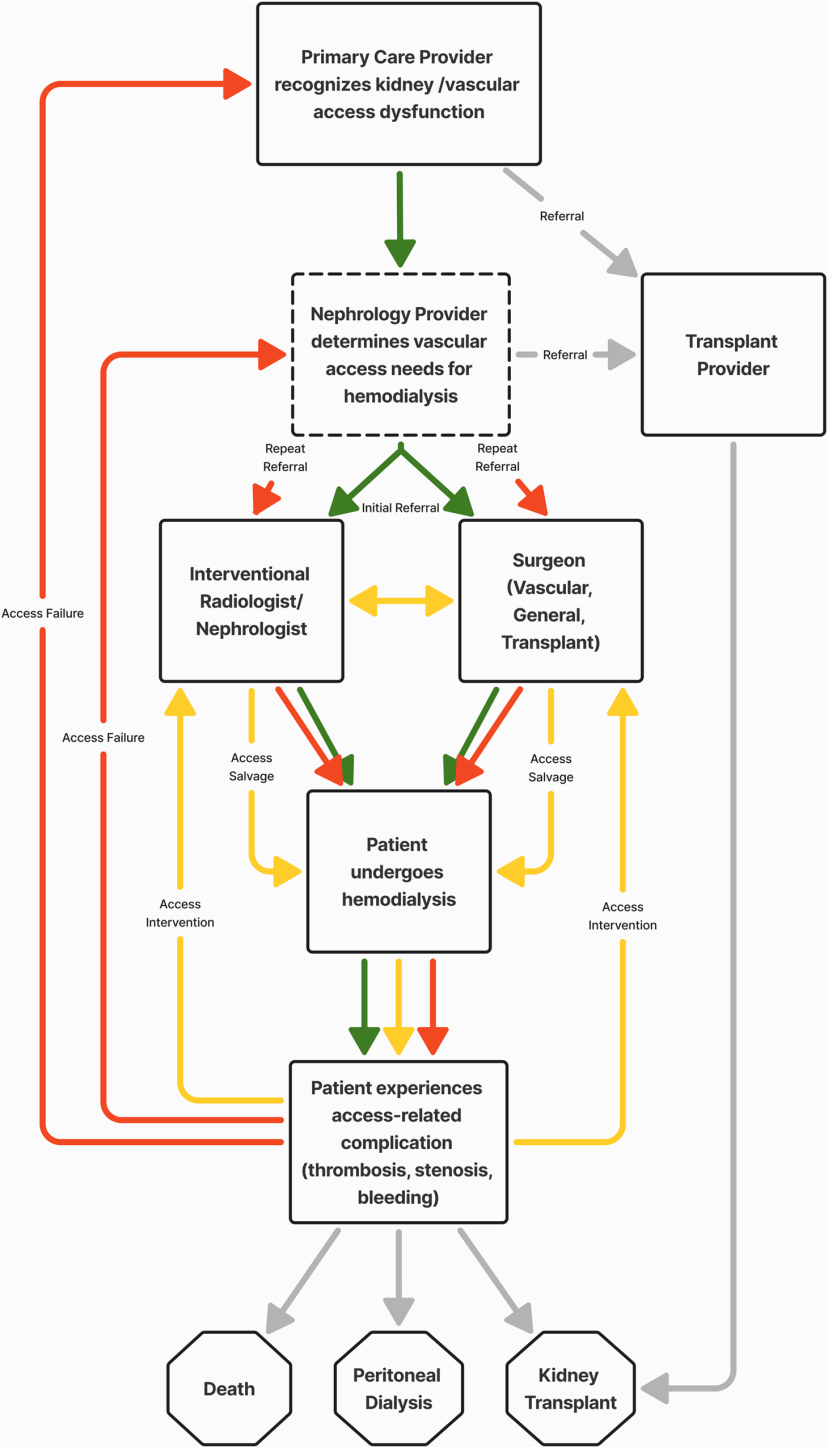
Stakeholder involvement in vascular access care and nonlinear trajectory of a patient with kidney failure requiring hemodialysis. The figure outlines the nonlinear trajectory of a patient with kidney failure requiring hemodialysis. Following initial referral to the nephrology provider, the patient begins on the path highlighted in green. The patient is referred to a surgeon for permanent access creation, interventional radiologist/nephrologist for tunneled line placement, or both, and then begins hemodialysis with their initial vascular access. Many patients will experience access-related complication(s) that prevents them from dialyzing safely. The patient must then enter the cycle highlighted in yellow in which they are evaluated by a surgeon and/or interventionalist and undergo procedure(s) for access salvage so they can continue to dialyze with their access. A patient may cycle through multiple rounds of access-related complications and interventions. Eventually, the patient’s access might fail completely, leading them to the path highlighted in red in which they must formulate a plan with their nephrology provider for their next vascular access, prompting repeat referrals to a surgeon and/or interventionalist. With each new access, the patient may continue to cycle through the yellow and red paths. Finally, they may eventually exit the cycle through kidney transplantation, transitioning to an alternative dialysis modality, or death

Vascular access planning primarily occurs between the patient and their nephrology team. Traditionally, these encounters tend to follow a paternalistic, access-centered approach with the nephrologist recommending a specific type of access after reviewing the patient’s medical history and comorbidities, risks and benefits, and referral to the relevant specialist for access creation [3]. In reality, vascular access care is a multistep process, reliant upon multidisciplinary expertise from multiple stakeholders to ensure appropriate access for hemodialysis, adding complexity to the upfront shared decision-making process. Additionally, the path from establishment of nephrology care to maintenance hemodialysis is not always as straightforward as patients and providers may hope. Following initial referral for vascular access, surgically created permanent access often takes months to establish maturity for successful cannulation and dialysis initiation. Furthermore, a significant proportion of patients undergo multiple interventions for access salvage and, should that access ultimately fail, patients must restart this arduous process from the beginning. As illustrated in Fig. 1, rather than a linear path from initial access creation to maintenance hemodialysis, many patients experience a cycle of access-related complications (e.g., thrombosis, stenosis, bleeding) and access interventions (e.g., thrombectomy, angioplasty, revision). Throughout this process, patients follow up not only with their nephrology providers (i.e., physicians, advanced practice providers, dialysis nurses and technicians), but also primary care providers, interventional radiologists and nephrologists, surgeons, and transplant providers to monitor current access function and proactively plan for the next option, including alternative access, peritoneal dialysis, kidney transplant, or withdrawal from dialysis.

Without effective interdisciplinary communication, care is destined to become fragmented and key information for shared decision-making, including the patient’s values and preferences, could be lost. For example, consider a patient who initiates hemodialysis with a tunneled central venous catheter and repeatedly declines referrals to see a vascular surgeon. They may be labeled a ‘difficult patient’ or a ‘refuser.’ Despite regular interactions with nephrology providers, dialysis staff, and social workers at the outpatient dialysis unit, none of the providers are able to elicit preferences against surgical evaluation. The patient’s longstanding primary care provider who manages their asthma and diabetes is unaware of efforts to transition to a permanent access. Had the primary care provider known, they would have shared that the patient’s mother died following surgery, leading to a strong preference against surgical interventions that could be further discussed. The siloing of information and care across primary and specialty providers limits the efficacy of shared decision-making between the patient and their providers. Improving interdisciplinary collaboration is necessary for effective implementation of shared decision-making. In this example, interprofessional collaboration ensures providers have a common understanding of the patient’s goals of care as informed by their values and concerns. Providers may then be able to engage the patient in more constructive shared decision-making by addressing specific concerns and making recommendations that align with their preferences.

Given the multidisciplinary nature of vascular access planning, a conventional shared decision-making model centered around the patient-physician dyad is insufficient. While nephrology providers have expertise in the longitudinal care of patients with kidney failure and are instrumental in identifying patients requiring dialysis, several other stakeholders bring specialized knowledge and unique experiences to the table that can significantly impact a patient’s preferences, care, and trajectory. For example, primary care providers have established longitudinal relationships with patients. Through routine healthcare visits, they monitor all the patient’s comorbid conditions, including kidney disease. They are well-positioned to identify when a nephrology referral is warranted, early signs of access dysfunction, and need for access intervention or replacement. Transplant providers determine not only whether a patient is a candidate for transplant, but also offer insight into the timeline to transplant, possibly influencing a patient’s decision about their vascular access. Additionally, general, vascular, and transplant surgeons who specialize in vascular access are trained in management of both vascular access (placement and revision) and vascular disease. Vascular surgeons, in particular, have broad expertise in both open and endovascular techniques and are the highest volume providers for vascular access creation [4, 5]. Interventional radiologists and nephrologists have expertise in endovascular establishment of vascular access and techniques for repair and revision. These proceduralists can provide objective assessments of a patient’s candidacy for each type of vascular access in the context of the patient’s medical history, comorbidities, and diagnostic modalities, such as vein mapping. Dialysis nurses and technicians are responsible for administering dialysis treatments, providing education, and addressing questions and concerns. From the significant time they spend with patients, these providers develop crucial insights into patient challenges and preferences related to vascular access. Social workers and patient navigators are essential for identifying and overcoming patient barriers to obtaining hemodialysis care, including difficulty navigating the healthcare system, lack of transportation, and unemployment. Finally, in the event a patient does not wish to initiate or continue dialysis, palliative care providers have critical expertise helping patients identify end-of-life goals and transitioning to conservative kidney management.

Interprofessional collaboration requires specialists across numerous disciplines to be unified in providing an integrated and cohesive approach to patient care [6]. An interprofessional approach to shared decision-making in vascular access planning has the potential to build upon the aforementioned conventional model of decision-making between patient and nephrology providers, which overlooks the inherently interprofessional nature of this complex intervention. We contend that an interdisciplinary, team-based approach to shared decision-making that includes nephrology providers, surgeons, interventional radiologists and nephrologists, primary care providers, transplant providers, dialysis nurses and technicians, social workers, patient navigators, and, most importantly, the patient will improve quality of communication, care continuity, clinical reasoning, incorporation of the patient’s values and preferences, and consensus [5, 7, 8]. Each team member brings critical expertise and perspectives that may significantly influence patient decisions related to vascular access. For example, in the case of a young patient expressing hesitation to their nephrologist about undergoing fistula creation because they believe a transplant will be available soon, a multidisciplinary discussion could inform the nephrologist that the patient has no viable living donors and has not yet been listed for transplantation, thereby allowing the nephrologist to set expectations with the patient regarding the timeline for a deceased donor transplant (i.e., up to 5 years on average) and engage in more nuanced shared decision-making.

We propose interdisciplinary team meetings to discuss access plans among nephrology providers, primary care providers, transplant providers (if applicable based on patient eligibility), surgeons, interventional radiologists and nephrologists, palliative care providers (if applicable based on patient goals), dialysis nurses and technicians, social workers, and patient navigators. Based on the Interprofessional Shared Decision-Making Model developed and validated by The Ottawa Hospital Research Group, the purpose of the meeting is to establish a common understanding of the most appropriate treatment options for a given patient and create a framework for shared decision-making that all providers would use with the patient and each other to ensure consistent communication of expectations, concerns, preferences, and plans [7]. This interprofessional approach has been applied in other complex medical care settings in and outside of the US, including kidney transplant selection committees [9], intensive care units [8], tumor boards [10], and cleft lip and palate programs [11]. For example, multiple parallels can be drawn between patients with kidney failure on hemodialysis and those with orofacial clefts: (1) the etiology of the disease is often multifactorial, (2) the disease often coincides with other diseases or syndromes, (3) the disease is chronic with complex clinical needs that require evaluations and treatments from an array of specialists over multiple decades of life. The American Cleft Palate-Craniofacial Association has published standards for the ideal composition, structure, and operation of a multidisciplinary team [12]. Similar evidence-based guidelines in other countries have been shown to improve patient care [11]. While the KDOQI recommends the use of a multidisciplinary team in the formulation of the End-Stage Kidney Disease Life-Plan, it does not offer specific guidance regarding its organization or function as there is currently no strong clinical evidence to inform implementation [1]. Although some US institutions already hold regular meetings to discuss patients with complex vascular access needs and have chronic kidney disease teams that help coordinate patient care and provide patient education, the degree to which non-nephrology providers are encouraged to participate is unclear and likely limited. Typically, additional specialists are involved only after a referral is placed by a nephrology provider or after development of a complication. Earlier inclusion of these team members at the time of initial access consultation may provide important insight regarding the likelihood of successful placement and maturation of permanent access [13], adding nuance to discussions regarding alternative access options. Finally, where multidisciplinary vascular access meetings already exist, clinical and research communities should be learning from them to understand participant composition, case discussions and prioritization, and implementation barriers and facilitators to help build the evidence base regarding their implementation and effectiveness with respect to decision-making and clinical outcomes.

The trajectory of a patient with kidney failure on hemodialysis is complex and dynamic; a patient’s access needs, personal values and preferences, comorbidities, and complications evolve over time, and the multidisciplinary team should similarly adapt to meet those needs by actively engaging all relevant providers in discussions regarding the patient’s vascular access care. The goal of interdisciplinary shared decision-making is not only to determine the most appropriate initial vascular access but also to plan proactively for future access needs, including interventions for salvage and alternative access creation should complications occur, all of which are essential for the formulation and maintenance of the End-Stage Kidney Disease Life-Plan. While a comprehensive, multidisciplinary approach to vascular access planning would require greater upfront investment in time by more providers, we anticipate downstream effects from more constructive discussions to determine the “right access in the right patient” may translate into improved patient selection, durability of access, access-related outcomes, access costs, care coordination, and alignment with patient preferences. Furthermore, Current Procedural Terminology codes for medical team conferences were created in 2008 to report time spent as part of a medical team discussing a patient’s care. Team conferences consisting of at least three different types of providers that have performed face-to-face evaluations and treatments outside of conference within the past 60 days meet criteria to use these codes [14, 15]. Providers, such as the multiple team members involved in vascular access planning, who care for patients on a regular basis and participate in complex discussions for 30 min regarding a patient’s care plan are thus able to bill for their additional efforts, offering an established incentive in the US to support interprofessional decision-making. Additionally, documentation of these multidisciplinary conferences allows not only the opportunity for providers to bill for their efforts, but also to record multidisciplinary team meeting summaries in the electronic medical record. Patients and their providers can then access information regarding the vascular access workup, recommended interventions, transplant candidacy, and more to help guide ongoing discussions and shared decision-making.

## Conclusions

More than 470,000 people with kidney failure in the US received hemodialysis in 2021 [16]. Dialysis treatment-related complications, including vascular access dysfunction, infections, and hemodynamic instability during dialysis, and the procedures and hospitalizations required to treat them are common and significantly impact patient quality of life [17]. The conventional model of shared decision-making centered on the nephrology-patient dyad is insufficient given the multidisciplinary nature of vascular access planning. Although currently there is no strong clinical evidence to guide implementation of a multidisciplinary team approach to vascular access care, this collaborative approach has been applied in other settings and shown to improve outcomes. Further research is critical to determine the structure, implementation, and effectiveness of interprofessional shared decision-making for vascular access planning. We argue for a comprehensive, interprofessional approach characterized by early and consistent collaboration among nephrology providers, primary care providers, transplant providers, surgeons, interventional radiologists and nephrologists, social workers, dialysis staff, patient navigators, and the patient. Each team member brings a critical perspective and unique expertise needed to identify the “right access, in the right patient, at the right time, for the right reasons.”

## Data Availability

No datasets were generated or analysed during the current study.

## Declarations

### Authors information

N/A.

## Abbreviations

AV: Arteriovenous
US: United States
KDOQI: Kidney Disease Outcomes Quality Initiative
